# Ketogenic therapy for schizophrenia: evidence, mechanisms, and clinical perspectives

**DOI:** 10.3389/fphar.2025.1603722

**Published:** 2025-06-25

**Authors:** Cristiano Chaves, Jennifer Fabe, Fabiano A. Gomes, Heather McNeely, Massimo Tusconi, Mauro Giovanni Carta, Serdar M. Dursun, Jaime E. C. Hallak, Elisa Brietzke

**Affiliations:** ^1^ NeuroMood Lab, Department of Psychiatry, School of Medicine and Kingston Health Sciences Centre (KHSC), Queen’s University, Kingston, ON, Canada; ^2^ Department of Psychiatry and Behavioural Neurosciences, McMaster University, Hamilton, ON, Canada; ^3^ Division of Pediatric Neurology, McMaster Children’s Hospital, Hamilton, ON, Canada; ^4^ Department of Medical Sciences and Public Health, University of Cagliari, Cagliari, Italy; ^5^ Department of Psychiatry (Neurochemical Research Unit) and Neuroscience and Mental Health Institute, University of Alberta, Edmonton, AB, Canada; ^6^ National Institute for Translational Medicine (INCT-TM), CNPq, São Paulo, Brazil; ^7^ Department of Neuroscience and Behavior, Ribeirão Preto Medical School, University of São Paulo, São Paulo, Brazil

**Keywords:** schizophrenia, ketogenic metabolic therapy (KMT), mitochondrial dysfunction, glucose metabolism, cognitive impairment, treatment resistance, neuroinflammation, adjunctive therapy

## Abstract

**Introduction:**

Schizophrenia is a chronic psychiatric disorder marked by significant cognitive and functional impairments. Current antipsychotic treatments offer limited benefit for negative symptoms and cognitive dysfunction while often exacerbating metabolic comorbidities. Emerging evidence implicates impaired glucose metabolism and mitochondrial dysfunction in the pathophysiology of schizophrenia, suggesting a role for metabolic interventions.

**Methods:**

This article reviews and synthesizes clinical, preclinical, and mechanistic evidence supporting the use of ketogenic therapy—a high-fat, low-carbohydrate intervention that induces ketosis—as a potential adjunctive treatment in schizophrenia.

**Results:**

Preliminary clinical findings, including case reports and small trials, suggest that ketogenic therapy may improve positive and negative symptoms, cognitive performance, and metabolic outcomes in individuals with schizophrenia spectrum disorders. Preclinical studies using NMDA antagonist models demonstrate that ketogenic interventions can normalize behavioral and neurophysiological deficits. Mechanistically, ketone bodies enhance mitochondrial function, modulate neurotransmitter systems (GABA, glutamate, dopamine), and reduce inflammation and oxidative stress. These effects may address core dysfunctions in schizophrenia that are unresponsive to dopamine-targeting pharmacotherapies.

**Discussion:**

Ketogenic therapy holds potential for addressing unmet clinical needs in schizophrenia, including negative and cognitive symptoms, treatment-resistant cases, and antipsychotic-induced metabolic syndrome. It may also be explored as a preventive strategy in high-risk populations. However, larger controlled trials are needed to establish efficacy, safety, and feasibility in psychiatric settings.

**Conclusion:**

Ketogenic therapy offers a novel, mechanistically informed intervention that targets metabolic and neurochemical pathways implicated in schizophrenia. If validated, it could pave the way for more integrative and personalized treatment strategies.

## 1 Introduction

Schizophrenia is a complex psychiatric disorder with positive (e.g., hallucinations), negative (e.g., social withdrawal), and cognitive symptoms. While current antipsychotics primarily target positive symptoms through dopamine modulation, they offer limited benefit for negative and cognitive symptoms, which are key drivers of long-term disability (Young and Geyer, 2015; Marder and Umbricht, 2023; Feber et al., 2025). This therapeutic limitation persists despite decades of advances in psychopharmacology and has left many patients with enduring impairments and serious treatment-related metabolic side effects (Henkel et al., 2022).

This ongoing failure points to a deeper, often unspoken problem in schizophrenia research: a dominant reliance on neurotransmission-based models, particularly dopaminergic dysfunction, which has shaped both our understanding of the disorder and the interventions developed to treat it. As a result, the research community remained locked into mechanisms that are insufficient for solving the most disabling aspects of the illness. Recent evidence challenge this narrow mechanistic lens by implicating metabolic dysregulation, including prefrontal cortical hypofunction and impaired glucose metabolism, as core pathophysiological features that may underlie these harder-to-treat symptoms (Henkel et al., 2022; Sethi and Ford, 2022; Omori et al., 2023).

These new findings highlight the importance of understanding how the brain’s energy demands and metabolic function may contribute to neuropsychiatric symptoms. The brain consumes about 20%–25% of the body’s resting glucose to support essential functions like synaptic transmission and neurotransmitter cycling (Aiello and Wheeler, 1995; Shulman et al., 2009). The neocortex, essential to cognition, is the most energy-demanding region, with high mitochondrial density and ATP production (Castrillon et al., 2023; Mosharov et al., 2025). Mitochondria are vital not only for energy production but also for neurotransmission and cell signaling (Picard and McEwen, 2018). In schizophrenia, disrupted glucose metabolism and mitochondrial dysfunction may contribute to oxidative stress, synaptic deficits, and cognitive impairments (Mitelman et al., 2018; Holper et al., 2019), supporting the potential role of metabolic interventions.

The most promising of these metabolic interventions is the ketogenic diet (KD), a high-fat, low-carbohydrate nutritional intervention used for over a century in epilepsy and now gaining attention as a neurometabolic therapy with broader neuropsychiatric potential (Ruskin et al., 2025; Shelp et al., 2025). By inducing ketosis and altering brain metabolism, KD may help improve psychiatric symptoms, including those in schizophrenia, by targeting underlying metabolic and neurochemical dysfunctions (Regenold et al., 2012; Sethi and Ford, 2022; Chrysafi et al., 2024).

Preliminary clinical evidence supports this therapeutic potential, with reports of KD leading to improvements in psychotic symptoms, mood stabilization, and metabolic health in individuals with schizophrenia and schizoaffective disorder. Although the literature remains limited, these early findings—comprising mostly case reports and a few small clinical studies—provide a compelling rationale for further investigation in controlled clinical settings.

The potential of KD lies in its unique metabolic mechanisms, which are increasingly being understood. The ketogenic diet shifts the brain’s energy source from glucose to ketone bodies, enhancing mitochondrial function and increasing ATP production. This metabolic boost may counteract cerebral glucose hypometabolism—a hallmark of schizophrenia (Du et al., 2014; Sullivan et al., 2019; Stein et al., 2023; Townsend et al., 2023; Chouinard et al., 2025). By improving brain energy metabolism, KD may restore prefrontal function and reduce the negative symptoms and cognitive deficits associated with hypofrontality (Hernandez et al., 2018; Henkel et al., 2022). The KD may also exert direct neuropharmacological effects by modulating neurotransmitter systems and neuroinflammation (Picard and McEwen, 2018; Daniels et al., 2020; Ji and Tang, 2025; Khatami et al., 2025).

This article reviews emerging clinical and preclinical evidence for ketogenic therapy in schizophrenia (Section 1), outlines the metabolic dysfunctions that support its rationale—such as glucose hypometabolism and mitochondrial impairment (Section 2), examines key neuropharmacological mechanisms (Section 3), and explores potential clinical applications across different stages and symptom domains (Section 4).

## 2 Current evidence supporting ketogenic therapy in schizophrenia

### 2.1 Clinical evidence: case reports and pilot trials

While still in its early stages, clinical research on the ketogenic diet (KD) in schizophrenia spectrum disorder is steadily gaining momentum. The available literature includes two small pilot trials, several case reports, and one retrospective analysis—all suggesting marked benefits across psychiatric and metabolic outcomes. Together, these findings underscore the need for a critical evaluation of the available clinical data.

Pacheco et al. (1965) conducted the first known pilot study of the ketogenic diet in schizophrenia, involving 10 women with chronic, treatment-resistant illness. Participants followed a ketogenic diet for just 2 weeks while continuing standard care. The authors reported a notable reduction in symptoms during the intervention, with partial relapse in most patients within a week of discontinuation. Major limitations included the brief duration, lack of ketone monitoring, and potential dietary non-adherence. Despite this preliminary evidence of potential benefit, this study remained largely overlooked and was not followed by replication or further investigation in the subsequent decades.

Almost 60 years later, the Pacheco et al. (1965) early trial was later echoed by a modern 4-month pilot study by Sethi et al. (2024), which enrolled 23 individuals (5 with schizophrenia, 18 with bipolar disorder) and comorbid metabolic dysfunction. While continuing usual psychiatric treatment, participants followed a ketogenic diet with nutritional support and ketone-based adherence tracking. Participants with schizophrenia on KD showed a 32% reduction in Brief Psychiatric Rating Scale (BPRS) scores. Metabolic improvements included reductions in weight (10%), waist circumference (11%), Body Mass Index (BMI) (10%), and visceral adipose tissue (27%). By the study’s end, no participants met criteria for metabolic syndrome. Although the sample included only five patients with schizophrenia, these preliminary findings support the feasibility and potential of ketogenic therapy as an adjunctive treatment.

An overview of the two pilot trials, as well as the published case reports and the sole retrospective cohort to date, is provided in Table 1.

**TABLE 1:** Summary of clinical studies evaluating the ketogenic diet in individuals with schizophrenia or schizoaffective disorder.

Study	Design	Sample Size	DX	Sex	Age (years old)	Duration	Main Findings	Metabolic Outcomes	Limitations	Ketosis Monitoring
Pacheco et al. (1965)	Open-label pilot trial	10	Schizophrenia (Chronic)	Female	19 to 63y	Two weeks	Significant decrease in symptomatology after 2 weeks; partial relapse after stopping the diet	Not systematically reported	No control group, small sample size, potential dietary nonadherence, short duration (2 weeks)	Not reported
Sethi et al. (2024)	Open-label pilot trial	5 with schizophrenia (23 whole cohort)	Schizophrenia (Chronic)	62% female (whole cohort)	43.4years (mean) ±15.6years (SD) (whole cohort)	4 months	32% reduction in BPRS scores in schizophrenia subgroup; improvements in CGI severity, life satisfaction, and sleep	12% weight loss; 13% BMI, 36% visceral adipose tissue, 27% HOMA-IR, and 25% triglyceride reductions; all with metabolic syndrome reversed​	Small schizophrenia subgroup, no control group, open-label design, self-selection bias	Yes; blood ketones measured weekly. 52.4% adherent (≥80% of readings in range). Higher adherence linked to better outcomes
Kraft and Westman (2009)	Case Report	1	Schizophrenia (Chronic)	Female	70y	12 months with remission maintained	Resolution of longstanding hallucinations and delusions after 8 days on the diet; no recurrence over 12 months	Weight loss (∼10 kg over 12 months); improved energy	Single case; no objective ketosis data; observational; dietary adherence self-reported	ketosis assumed based on diet but not measured
Palmer (2017)	Case Series	2	Schizoaffective Disorder (Chronic)	Female (Case 1) and Male (Case 2)	33years (Case 1) and 31 years (Case 2)	12 months (Case 1), 4 months (Case 2)	PANSS scores dropped from 98 to 49 (pos: 27→13, neg: 25→8) and 107 to 70 (pos: 24→15, neg: 29→18); symptoms returned off diet and improved again with resumption	Significant weight loss in both cases (104 lb and 30 lb); improved energy and functioning	Only two cases; limited generalizability	Confirmed by ketone urinanalysis
Gilbert-Jaramillo et al. (2018)	Case Series	2	Schizophrenia (Chronic)	Female (Case 1) and Male (Case 2)	2 opposite-sex twins	6 weeks (∼2 weeks on ketosis)	Modest PANSS score reductions (female: 101→91; male: 82→75); symptoms returned post-diet	Reduction in body fat in both cases; normalization of liver enzymes in male patient	Only two cases; poor adherence; short ketosis duration	Confirmed by ketone urinanalysis
Palmer et al. (2019)	Case Series	2	Schizophrenia (Chronic)	Female	82years (Case 1) and 39 years (Case 2)	12 years (Case 1) and 5 years (Case 2)	Sustained full remission for 5 and 12 years on KD without antipsychotics	150 lb weight loss in one case; regained independence	Only two cases; self-reported adherence; no ketone data; retrospectice report	Not reported
Laurent et al. (2025)	Case Series	2	Schizoaffective Disorder (Chronic)	Female	17years (Case 1) and 32 years (Case 2)	24 weeks (Case 1), 52 weeks (Case 2)	Full remission of psychotic and mood symptoms; functional recovery and reduced medications	Reduction in body fat with mild or no weight loss	Case series; limited generalizability; no long-term follow-up	Yes (BHB levels measured). Blood Ketone levels between 0.8-3.5 (case 1) and 3.3 mmol/L (not consistently measured afterwards)
Danan et al. (2022)	Retrospective analysis	10 with schizoaffective disorder (31 whole cohort)	Schizoaffective Disorder (Chronic)	71% female (whole cohort)	50years (mean) ± 11.3years (SD) (whole cohort)	15–248 days (mean ∼59 days)	PANSS scores (n = 10) decreased from 91.4 to 49.3; subscale scores not reported	48% lost ≥5% body weight; improvements in blood pressure, fasting glucose, HbA1c, GGT, cholesterol, and triglycerides	No control group, limited ketosis data, semi-controlled setting, retrospective design	Urine ketones measured once; 64% tested positive

As summarized in Table 1, several case reports have also documented notable psychiatric improvements in schizophrenia and schizoaffective disorder. Firstly, Kraft and Westman (2009) described a 70-year-old woman with chronic schizophrenia who experienced complete remission of daily hallucinations within 8 days of starting KD. Her symptoms remained in remission for over a year, with brief relapses during dietary lapses. This case is the same individual later reported in Palmer et al. (Palmer et al., 2019), where sustained remission was documented over a 12-year follow-up.

Palmer (2017) also described two cases of schizoaffective disorder in which patients experienced substantial improvements in both positive and negative symptoms while following a ketogenic diet.

The first case involved a 33-year-old man with long-standing, treatment-resistant symptoms. His baseline PANSS score was 98 (positive = 27, negative = 25, general = 46). After starting the ketogenic diet, he reported rapid symptom improvement, including reduced hallucinations and delusions, and better mood, energy, and concentration. After one year—and a 104-pound weight loss—his PANSS score dropped to 49, with negative symptoms falling to 8, just above the minimum score of 7. This near-remission represents a floor effect and is notable given the lack of effective treatments for negative symptoms. Symptom relapses occurred when he broke the diet and consistently resolved upon resuming ketosis.

The second case was a 31-year-old woman with similarly refractory symptoms, unresponsive to multiple medications and electroconvulsive therapy. Her baseline PANSS score was 107 (positive = 24, negative = 29, general = 54). After 4 months on the ketogenic diet and 30 pounds of weight loss, her score improved to 70 (positive = 15, negative = 18, general = 38). A return of persecutory delusions followed dietary discontinuation and did not respond to an increased aripiprazole dose but resolved only after a 3-day fast that restored ketosis.

Another case report by Gilbert-Jaramillo et al. (2018) described possible clinical benefits of short-term ketosis despite poor adherence. The authors followed 22-year-old opposite-sex twins with schizophrenia over 6 weeks on a ketogenic diet. Both had periods of non-compliance and achieved significant ketosis for only about 2 weeks. During this time, PANSS scores showed modest improvements (female: 101 to 91; male: 82 to 75), and both experienced reductions in body fat. Psychiatric symptoms partially returned after discontinuing the diet, consistent with previous observations that ketosis may mediate symptom improvement.

In a later report, Palmer et al. (2019) retrospectively described two cases of chronic schizophrenia in which patients achieved sustained remission of psychotic symptoms while following a ketogenic diet and discontinuing antipsychotic medications. An 82-year-old woman experienced rapid improvement after starting the diet at age 70, remained symptom-free, and lived independently without relapse for 12 years. A 39-year-old woman achieved full remission within a month of starting the diet, successfully tapered off antipsychotics, and remained stable for 5 years. Although standardized symptom scales were not used, these cases suggest the ketogenic diet may contribute to lasting remission in treatment-resistant schizophrenia.

In a larger retrospective analysis, Danan et al. (2022) examined 31 psychiatric inpatients with treatment-resistant illness, including 10 with schizoaffective disorder, who followed a medically supervised ketogenic diet. Among those with schizoaffective disorder, total PANSS scores improved significantly (from a mean of 91.4 to 49.3), although subscale scores were not reported. Metabolic outcomes also improved, with reductions in weight (48% of those on antipsychotics lost ≥5%), blood pressure, fasting glucose, HbA1c, GGT, cholesterol, and triglycerides. Urine ketones were measured at least once per participant to confirm ketosis, though monitoring was limited due to the clinical setting. The intervention was well tolerated, demonstrating both feasibility and preliminary efficacy in a psychiatric inpatient setting.

More recently, Laurent et al. (2025) reported two cases of schizoaffective disorder achieving full remission of psychotic and mood symptoms through a medically supervised ketogenic diet. The first case, a 17-year-old female with treatment-resistant illness, stabilized within 6 weeks and maintained improvement over 24 weeks, as measured by standard measures for anxiety (GAD-7), depression, anxiety, and stress (DASS-42), and PTSD (PCL-5), though no schizophrenia-specific scale was used. She reduced her medication to a single psychotropic without experiencing relapse. The second case, a 32-year-old female with chronic psychosis, cognitive impairment, and a history of multiple suicide attempts, achieved full remission within 6 months, maintained ketosis for a year, and discontinued all medications without recurrence. While the lack of schizophrenia-specific symptoms outcomes limits interpretation, these cases highlight the potential of ketogenic metabolic therapy for severe, treatment-resistant schizoaffective disorder.

In review of the clinical evidence, the ketogenic diet therapy approaches are variable in description. It was often presumed that the ketogenic diet intervention was the reason for the ketosis being achieved. Caloric analysis is not well reported in these studies. We should be mindful that ketosis could have been achieved with sub-optimal caloric intake also. When considering compliance, we need to be further mindful that sensations of hunger may affect adherence. There is likely starvation physiology intermingled with intentional ketogenic diet metabolic therapies.

Although some studies described the type of foods recommended, the actual macronutrient intake needed further characterization. Macronutrient description is needed to actually appreciate the consistent shift of fat, protein and carbohydrate substrates to favor ketosis.

Finally, evidence further suggests that compliance to diet therapy requires counselling before and during the intervention. Customizing the complex KD intervention to the patient’s clinical, psychological and social circumstances is an important counselling strategy that should be also considered when evaluating reasons for adherence or lack thereof (Kalra et al., 2019).

Despite these limitations, the reports suggest that the ketogenic therapy may improve both psychiatric and metabolic outcomes in individuals with schizophrenia spectrum disorders. Although preliminary, these findings highlight a promising therapeutic avenue and emphasize the need for larger, well-controlled clinical trials to establish efficacy and guide clinical practice.

### 2.2 Preclinical insights: animal models of schizophrenia

A small but growing body of preclinical research has investigated the effects of ketogenic interventions in animal models of schizophrenia, in particular those involving NMDA receptor hypofunction. Five key studies have examined the ketogenic diet (KD) or its principal metabolite, β-hydroxybutyrate (BHB), across various schizophrenia-relevant behavioral and neurophysiological endpoints.

In a foundational study, Kraeuter et al. (2015) demonstrated that a ketogenic diet administered for 3 weeks reversed behavioral abnormalities induced by the NMDA receptor antagonist MK-801 in male C57BL/6 mice. Specifically, KD attenuated MK-801-induced hyperactivity, stereotyped behavior, and ataxia—features analogous to positive symptoms. Additionally, it normalized social withdrawal and spatial working memory deficits, representing negative and cognitive symptom domains, respectively. These effects occurred alongside significant metabolic shifts (elevated β-hydroxybutyrate and reduced glucose), suggesting potential mechanisms involving altered energy metabolism and neurotransmitter modulation​.

Building on this work, Kraeuter et al. (2019b) examined whether KD could prevent deficits in prepulse inhibition (PPI), a translational measure of sensorimotor gating impaired in schizophrenia. KD effectively prevented MK-801-induced PPI deficits at both three and 7 weeks, independent of caloric intake or weight loss, supporting the notion that its effects are metabolically driven rather than due to energy restriction​.

In a follow-up study using female mice, Kraeuter et al. (2019a) compared the effects of KD and the antipsychotic olanzapine on MK-801-induced PPI disruption. Both KD and olanzapine independently mitigated PPI deficits, with no additive effect when combined. These findings indicate that KD may be as effective as antipsychotic in modulating sensorimotor gating​, supporting its potential as a standalone or adjunctive therapy.

Tregellas et al. (2015) extended the investigation to a genetically predisposed model using DBA/2 mice, which exhibit naturally impaired hippocampal P20/N40 auditory gating—an electrophysiological correlate of the human P50 deficit observed in patients with schizophrenia. Mice on KD showed significant improvements in auditory gating, exceptionally those with higher blood ketone levels. These findings suggest enhanced hippocampal inhibitory function, a key target in schizophrenia pathophysiology.

Finally, Kraeuter et al. (2020) explored whether exogenous BHB administration alone could replicate the therapeutic effects of KD. Acute high-dose BHB attenuated MK-801-induced hyperlocomotion and PPI deficits, while chronic BHB treatment over 3 weeks normalized behavioral abnormalities across positive (hyperactivity), negative (social withdrawal), and sensorimotor gating domains. These results highlight the role of BHB as a key mediator of KD’s effects and point to the possibility of using exogenous ketones as a practical alternative to strict dietary interventions.

A summary of the key characteristics and findings from these five preclinical studies is presented in Table 2.

**TABLE 2:** Summary of preclinical studies on ketogenic therapy in animal models of schizophrenia.

Study	Model	Intervention	Sample Size	Main Findings
Kraeuter et al. (2015)	MK-801-induced schizophrenia-like behavior in male C57BL/6 mice	Ketogenic diet or Standard diet (3 weeks)	8 per group	KD reversed MK-801-induced hyperactivity, stereotypy, social withdrawal, and working memory deficits
Kraeuter et al. (2019b)	MK-801-induced PPI deficits in male C57BL/6 mice	Ketogenic diet or Standard diet (3 weeks)	8 per group	KD prevented PPI deficits induced by MK-801. Effects independent of calorie restriction or body weight
Kraeuter et al. (2019a)	MK-801-induced PPI deficits in female C57BL/6 mice	KD (6 months), Olanzapine (8 weeks), alone and in combination	13 per group	KD, olanzapine, and their combination reversed MK-801-induced PPI deficits; KD alone comparable to antipsychotic
Tregellas et al. (2015)	P20/N40 auditory gating deficits in DBA/2 male mice	Ketogenic diet (3 weeks)	12 total (6 low, 6 high ketosis)	KD improved P20/N40 auditory gating; ketone levels correlated with better gating performance
Kraeuter et al. (2020)	MK-801 model with Beta-hydroxybutyrate (BHB) administration	BHB injections - acute and chronic (3 weeks)	Acute: n = 8–24/group, Chronic: n = 24/group	Acute BHB suppressed MK-801 effects; chronic BHB normalized hyperlocomotion, social withdrawal, and PPI deficits

As summarized in Table 2, these preclinical findings suggest that ketogenic interventions, either through diet or exogenous ketone supplementation, may ameliorate multiple behavioral and neurophysiological deficits associated with schizophrenia. The consistent cognitive and behavioral improvements in animal models, likely mediated by metabolic and neurotransmitter changes, underscore the potential of ketogenic therapy and support further research into underlying mechanisms and clinical translation.

## 3 Metabolic pathophysiology of schizophrenia: a rationale for ketogenic therapy

**FIGURE 1:**
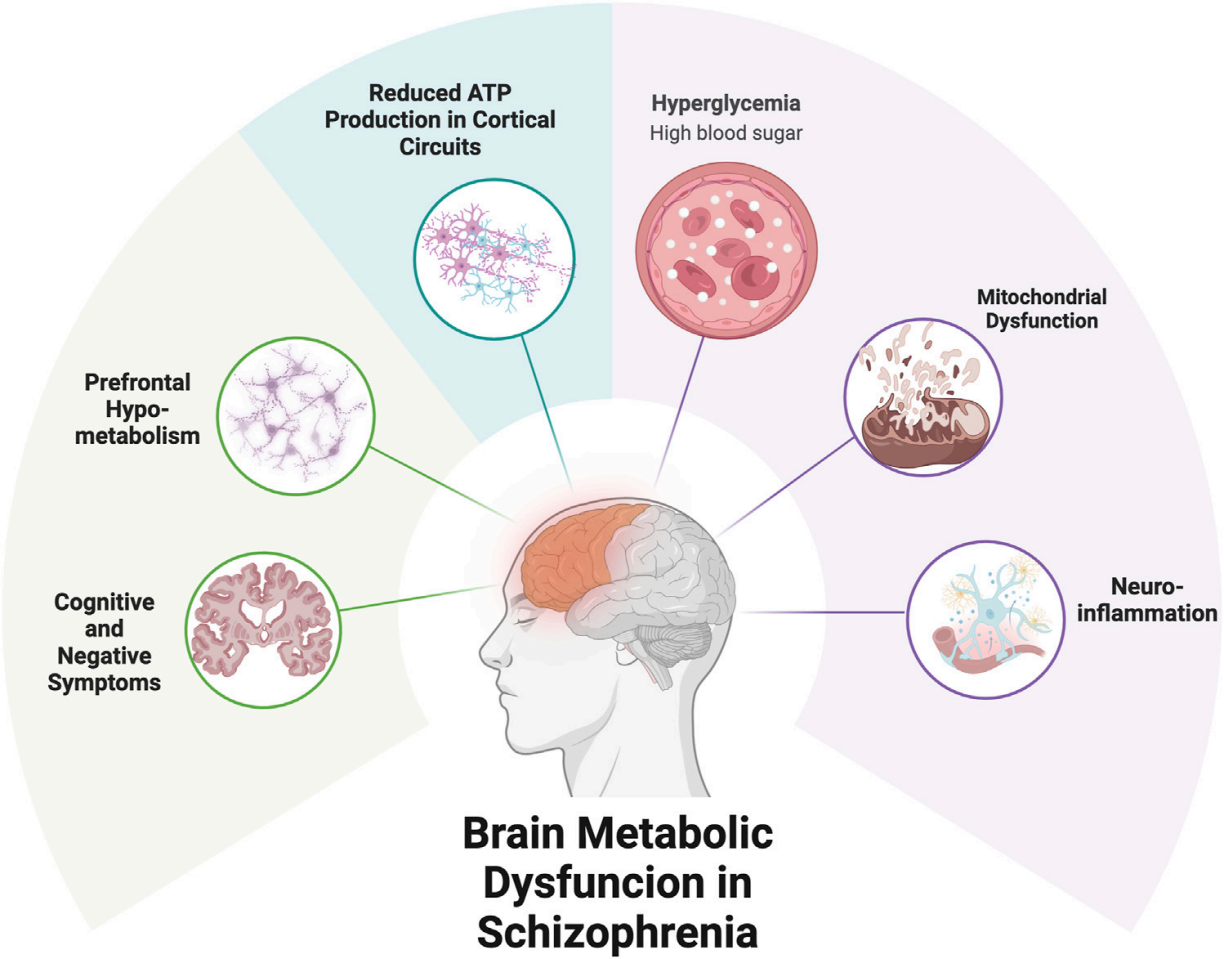
Metabolic Dysfunction and Symptom Expression in Schizophrenia. Caption: Metabolic impairments in schizophrenia may contribute to reduced energy availability in cortical circuits, particularly the prefrontal cortex. Mitochondrial dysfunction and impaired glucose utilization can limit ATP production, leading to synaptic instability and functional deficits. These disruptions may underlie negative symptoms and cognitive impairments. Ketogenic therapy offers a strategy to bypass glucose-dependent pathways and support brain energy metabolism via ketone bodies.

Growing evidence suggests that schizophrenia involves not only neurotransmitter dysregulation but also fundamental impairments in energy metabolism, including disrupted glucose utilization and mitochondrial dysfunction. These abnormalities may underlie core clinical features—particularly cognitive deficits and negative symptoms—and are not targeted by current pharmacological treatments. Box 1 and Figure 1 provide an overview of brain energy demands and how their disruption may contribute to symptom expression. The following subsections examine schizophrenia-specific evidence for glucose metabolism impairment, brain energy deficits, and mitochondrial dysfunction, and highlight the limitations of existing treatments in addressing these pathophysiological domains.

Box 1 **| Why Brain Energy Matters in Schizophrenia.**

• The brain consumes ∼20–25% of the body’s resting glucose, despite being only ∼2% of body weight (Aiello and Wheeler, 1995; Shulman et al., 2009).
• High-order cortical regions like the prefrontal cortex are especially energy-demanding due to dense synaptic activity (Mosharov et al., 2025).
• These regions are rich in neuromodulatory receptors and G-protein-coupled signaling pathways, making them vulnerable to metabolic stress (Castrillon et al., 2023).
• In schizophrenia, cognitive and negative symptoms may reflect impaired energy supply to these circuits (Henkel et al., 2022; Sullivan et al., 2019).
• This energy vulnerability supports the rationale for metabolic interventions such as ketogenic therapy (Sethi and Ford, 2022; Regenold et al., 2012).

### 3.1 Glucose metabolism impairment in schizophrenia

Evidence accumulated over the past 2 decades consistently indicates that individuals with schizophrenia, even at illness onset and prior to antipsychotic exposure, show significant impairments in glucose metabolism. Several studies demonstrate that these abnormalities are not solely attributable to medication effects or lifestyle factors, but may instead reflect intrinsic metabolic alterations associated with the disorder (Pillinger et al., 2017).

A seminal study by Ryan et al. (2003) found that 15% of drug-naive, first-episode patients with schizophrenia met criteria for impaired fasting glucose, while none of the matched healthy controls did. These patients also showed higher fasting glucose, insulin, cortisol levels, and increased insulin resistance as measured by Homeostatic Model Assessment of Insulin Resistance (HOMA-IR), independent of body composition or lifestyle variables​.

Subsequent studies confirmed these findings. Steiner et al. (2017) reported significantly elevated insulin resistance and fasting insulin levels in first-episode schizophrenia patients, accompanied by increased cortisol and catecholamine metabolites, suggesting a stress-related contribution to metabolic dysfunction​. Notably, the increase in HOMA-IR was not fully explained by body mass index, cortisol levels, or smoking, indicating disease-specific alterations in glucose homeostasis.

In a rigorous meta-analysis, Perry et al. (2016) reviewed data from antipsychotic-naive individuals with first-episode psychosis and found increased rates of impaired glucose tolerance and insulin resistance, though not of fasting plasma glucose *per se*​. These results imply that glucose dysregulation may already be present in a subclinical form at early disease stages.

Further supporting these observations, Pillinger et al. (2017) synthesized data from 16 case-control studies and found that antipsychotic-naive patients with first-episode schizophrenia had significantly higher fasting glucose, post-challenge glucose, insulin levels, and insulin resistance compared to controls. Importantly, this meta-analysis used standardized methods to control for confounding factors including BMI and diet​.

In a complementary investigation, Fernandez-Egea et al. (2009) found that individuals with non-affective psychosis had a significantly higher prevalence of abnormal glucose tolerance, as measured by oral glucose tolerance tests, compared to matched controls. This metabolic dysfunction was present despite controlling for cortisol, BMI, smoking, and socioeconomic status​.

Genetic evidence also suggests a shared biological basis for schizophrenia and type 2 diabetes. Hackinger et al. (2018) demonstrated that individuals with both schizophrenia and diabetes carry a higher genetic risk burden for both conditions. Several genomic loci were found to colocalize between schizophrenia and diabetes, further supporting the notion of overlapping pathophysiological pathways​.

### 3.2 Brain energy deficits in schizophrenia

Beyond peripheral glucose dysregulation, there is compelling evidence of intrinsic cerebral energy deficits in schizophrenia. These deficits manifest as mitochondrial dysfunction, reduced efficiency of ATP production, abnormal lactate accumulation, and altered bioenergetic enzyme activity—primarily within prefrontal brain regions.

One of the most direct markers of impaired brain bioenergetics is elevated cerebral lactate, a byproduct of anaerobic glycolysis. Rowland et al. (2016), using ultra-high field 7T magnetic resonance spectroscopy (MRS), demonstrated that individuals with schizophrenia exhibited significantly higher lactate concentrations in the anterior cingulate cortex compared to controls. Earlier electrophysiological (ERP) research had already demonstrated impaired error monitoring during a cognitive inhibition task in adults with schizophrenia versus health controls that localized to anterior cingulate dysfunction in the patient sample (Alain et al., 2002). In the Rowland et al. (2016) sample, elevated lactate correlated with both cognitive deficits and impaired functional capacity, suggesting a shift from oxidative phosphorylation to less efficient energy metabolism pathways in schizophrenia​.

More recent MRS findings support a progressive bioenergetic shift over the course of illness. Stein et al. (2023) reviewed evidence from longitudinal MRS studies, showing that both first-episode and chronic schizophrenia patients exhibit reduced creatine kinase (CK) activity and redox imbalance in the prefrontal cortex. Chronic patients also show increased lactate levels and decreased intracellular pH, consistent with a shift from oxidative phosphorylation toward glycolysis. These deficits in ATP regeneration, energy buffering, and redox regulation may emerge early and intensify over time, potentially contributing to persistent cognitive dysfunction.

Further supporting mitochondrial involvement, Du et al. (2014) found a 22% reduction in creatine kinase forward rate constant in the frontal lobe using phosphorus-31 magnetization transfer spectroscopy. This reflects impaired ATP buffering capacity and suggests decreased mitochondrial energy flux. Additionally, a lower intracellular pH in patients pointed to increased reliance on glycolysis—a hallmark of inefficient energy metabolism​.

Sullivan et al. (2019) expanded on this view by reporting neuron-specific reductions in key glycolytic and glucose transport enzymes in the dorsolateral prefrontal cortex (DLPFC), including hexokinase, phosphofructokinase, and glucose transporters GLUT1 and GLUT3. These changes were not attributable to antipsychotic use and were specific to pyramidal neurons, indicating that schizophrenia may involve cell-type-specific energy deficits that directly impair synaptic and cognitive function​.

At the ultrastructural level, Hara et al. (2014) demonstrated that the morphology of presynaptic mitochondria in the prefrontal cortex correlates with cognitive performance. In primates, greater working memory capacity was associated with a higher proportion of straight, healthy mitochondria, while impaired performance was linked to donut-shaped mitochondria, which are associated with metabolic stress. This structural vulnerability may help explain how mitochondrial health contributes to functional brain integrity​.

Compelling evidence from PET imaging studies further confirms cerebral energy deficits in schizophrenia. Townsend et al. (2023) conducted a meta-analysis of 36 [^18^F]FDG-PET studies and found significantly reduced glucose metabolism in the frontal cortex of individuals with schizophrenia. Specifically, absolute glucose metabolism in the frontal lobes was significantly lower in patients compared to healthy controls (Hedges’ g = −0.74), and frontal metabolism relative to whole-brain glucose uptake was also decreased (Hedges’ g = −0.44). These findings highlight both a general reduction in frontal brain energy use and a disproportionate impact on this region compared to the rest of the brain. The authors found no consistent abnormalities in other cortical regions, underscoring the regional specificity of frontal energy dysfunction and its potential contribution to negative and cognitive symptoms.

Most recently, Chouinard et al. (2025) showed that cognitive impairment in psychotic disorders is associated with decreased creatine kinase activity and increased reductive stress, as reflected by a lower NAD+/NADH ratio. These findings further suggest that energy metabolism dysfunction may be a correlate of the cognitive burden in psychotic illnesses.

### 3.3 Mitochondrial dysfunction in schizophrenia

Mitochondria are central regulators of cellular energy production, biosynthesis, and apoptotic signaling, making them especially critical in high-demand tissues like the brain. An expanding body of research implicates mitochondrial dysfunction in the pathophysiology of schizophrenia, driven both by genetic factors and environmental stressors across the lifespan.

Meta-analytic evidence confirms reductions in the activity of mitochondrial complex I and, to a lesser extent, complex IV in individuals with schizophrenia. Holper et al. (2019) demonstrated moderate effect sizes for reduced complex I activity in the frontal cortex, striatum, and cerebellum of patients with schizophrenia, a pattern distinct from the more pronounced mitochondrial dysfunction observed in neurodegenerative disorders such as Parkinson’s or Alzheimer’s disease​. These enzyme abnormalities suggest inefficient oxidative phosphorylation and impaired ATP generation in key brain circuits in patients with schizophrenia and related disorders.

Stress and early-life adversity are increasingly recognized as key environmental contributors to mitochondrial vulnerability. Mitochondria are highly dynamic organelles that respond to neuroendocrine stress mediators such as glucocorticoids and catecholamines. Picard and McEwen (2018) proposed that mitochondria not only produce the energy required for stress adaptation but are themselves affected by chronic stress, leading to altered energy metabolism, oxidative stress, and downstream dysfunction in neuroplasticity and behavior​. Daniels et al. (2020) further reviewed how psychosocial stress may recalibrate mitochondrial structure and function in a maladaptive direction, thereby linking environmental adversity to long-term risk for psychiatric disorders​.

This is especially relevant in schizophrenia, where cumulative environmental exposures—including childhood trauma, cannabis use, and minority immigration status—have been shown to increase disease liability, as captured by the Maudsley Environmental Risk Score (Vassos et al., 2020). These risk factors are also associated with cognitive impairment at psychosis onset. In a large first-episode psychosis cohort, higher environmental risk scores predicted poorer cognitive profiles and greater premorbid deterioration (Ferraro et al., 2025). Such findings suggest that environmental stressors may impact brain development and cognition through mitochondrial dysregulation, providing a mechanistic bridge between adversity and the emergence of cognitive and negative symptoms in schizophrenia.

Regenold et al. (2012) identified a specific mitochondrial alteration in postmortem brain tissue from patients with schizophrenia: detachment of hexokinase 1 (HK1) from the outer mitochondrial membrane. This detachment disrupts the coupling between glycolysis and oxidative phosphorylation, reducing ATP production and increasing reliance on inefficient metabolic pathways such as the polyol pathway, thus impairing both energy metabolism and neuronal resilience​.

One biomarker reflecting shifts in cerebral energy metabolism is β-hydroxybutyrate (BHB), the principal ketone body used as an alternative fuel source when glucose metabolism is impaired. Several studies report altered BHB levels in individuals with schizophrenia. Yang et al. (2013) found significantly elevated BHB and pyruvate in both serum and urine of schizophrenia patients compared to healthy controls, suggesting a compensatory upregulation of fatty acid oxidation in the context of cerebral glucose hypometabolism​. Similarly, Huang et al. (2016) reported higher serum BHB levels in schizophrenia patients even after controlling for metabolic confounders, while pyruvate was elevated only in subgroups treated with olanzapine or clozapine​. In a follow-up prospective study, Huang et al. (2018) found that BHB levels decreased over the course of antipsychotic treatment, and that changes in BHB were positively correlated with executive function improvements, raising the possibility that BHB may play a protective or modulatory role in cognitive function​.

These findings suggest that mitochondrial dysfunction in schizophrenia is multifaceted—involving direct impairments in the electron transport chain, disrupted metabolic coupling, and maladaptive responses to environmental stress. The frequent observation of elevated BHB in schizophrenia may reflect a compensatory response to these deficits and supports further investigation into ketone-based interventions.

### 3.4 Limitations of current treatments in addressing energy dysfunction

While current antipsychotics remain the cornerstone of schizophrenia treatment, their mechanism of action primarily targets dopaminergic signaling and offers limited impact on the underlying bioenergetic deficits. These agents are effective in reducing positive symptoms but provide minimal benefit for negative symptoms, cognitive impairment, or functional recovery—domains increasingly associated with impaired brain energy metabolism (Young and Geyer, 2015; Henkel et al., 2022; Marder and Umbricht, 2023; Chouinard et al., 2025; Feber et al., 2025).

Moreover, antipsychotics, especially second-generation agents, often exacerbate metabolic disturbances. Treatment-related weight gain, insulin resistance, dyslipidemia, and increased visceral adiposity contribute to the markedly reduced life expectancy in schizophrenia and further burden cognitive and functional outcomes (Henkel et al., 2022). This metabolic toxicity is especially concerning given the emerging evidence of pre-existing mitochondrial dysfunction and glucose metabolism abnormalities in schizophrenia, even prior to pharmacological intervention.

Despite advances in neuroscience and pharmacology, no currently approved treatments directly address the brain’s energy demands or restore mitochondrial function. Cognitive enhancers, glutamatergic agents, and anti-inflammatory compounds have shown mixed results in clinical trials and have not translated into routine care (Howes et al., 2024; Horan et al., 2025). These limitations underscore the need for therapeutic strategies that target the cellular and metabolic underpinnings of the disorder.

In this context, interventions that can restore mitochondrial function, reduce reliance on glucose metabolism, and support alternative energy pathways—such as ketogenic therapy—warrant further investigation. By bypassing impaired glycolytic flux and enhancing ATP production through ketone metabolism, ketogenic interventions offer a novel route to address the energetic deficits increasingly recognized as central to schizophrenia pathophysiology.

## 4 Neurobiological mechanisms of ketogenic therapy

The ketogenic diet (KD), by shifting the brain’s primary energy source from glucose to ketone bodies, exerts a range of neurobiological effects with potential relevance to schizophrenia. Originally used to treat epilepsy, KD has demonstrated influence across multiple domains—mitochondrial function, neurotransmission, and inflammation—all of which are implicated in schizophrenia pathophysiology.

### 4.1 Restoration of mitochondrial function and brain energy metabolism

A key mechanism underlying the therapeutic potential of ketogenic therapy is its capacity to restore mitochondrial function and improve brain energy metabolism. Ketogenic diets induce a metabolic state in which ketone bodies—primarily BHB—serve as the main energy substrates, bypassing glycolytic pathways that may be impaired in schizophrenia. This shift toward fatty acid oxidation and ketone metabolism enhances mitochondrial efficiency and promotes multiple adaptive responses at the cellular level.

Ketone bodies provide a more efficient fuel source for the brain compared to glucose, producing more ATP per molecule of substrate and generating less reactive oxygen species (ROS) during oxidative phosphorylation​ (Miller et al., 2018). The entry of ketone bodies into mitochondria facilitates acetyl-CoA production and supports the tricarboxylic acid (TCA) cycle even under conditions of glucose hypometabolism, a hallmark of schizophrenia. This compensatory energy mechanism is especially relevant given the widespread mitochondrial deficits observed in patients.

Ketogenic therapy also enhances mitochondrial biogenesis and dynamics. The KD activates AMP-activated protein kinase (AMPK) and increases the expression of peroxisome proliferator-activated receptor gamma coactivator 1-alpha (PGC-1α), a master regulator of mitochondrial biogenesis (Kyriazis et al., 2022)​. This promotes transcription of genes involved in oxidative phosphorylation and fatty acid oxidation, boosting mitochondrial quantity and quality in high-demand tissues such as the brain.

Further, ketone bodies such as BHB act as signaling molecules, inducing epigenetic and antioxidant effects. BHB can inhibit histone deacetylases (HDACs), leading to increased expression of antioxidant and mitochondrial-protective genes (Williams et al., 2024)​. BHB also contributes to redox homeostasis by stimulating the expression of antioxidant enzymes, including superoxide dismutase and catalase (Miller et al., 2018)​. This phenomenon, known as *mitohormesis*, represents a mild cellular stress that promotes long-term adaptive resilience against oxidative damage and mitochondrial dysfunction.

Ketogenic interventions may also influence mitochondrial quality control mechanisms. They appear to support balanced mitochondrial fission and fusion, and enhance mitophagy, the process of selectively removing damaged mitochondria to maintain organelle integrity (Kyriazis et al., 2022)​. These effects further ensure metabolic stability and reduce vulnerability to stress-induced cellular injury.

Collectively, these mechanisms suggest that ketogenic therapy not only compensates for impaired glucose metabolism but also restores mitochondrial function and energy availability through both direct bioenergetic support and long-term molecular adaptations. Such effects are distinctly promising in schizophrenia, a disorder characterized by widespread brain energy deficits, mitochondrial dysfunction, and oxidative stress.

### 4.2 Modulation of neurotransmission: GABA, glutamate, and dopamine

Beyond its metabolic effects, the ketogenic diet (KD) also modulates key neurotransmitter systems involved in schizophrenia. Disruptions in excitatory-inhibitory (E/I) balance—particularly those affecting glutamate, GABA, and dopamine—are central to the disorder’s pathophysiology. In schizophrenia, this imbalance is often marked by diminished GABAergic function and excessive glutamatergic activity, especially within the prefrontal cortex and hippocampus (Howes et al., 2024). Emerging evidence suggests that KD may help restore E/I balance by enhancing inhibitory signaling, dampening glutamate release, and indirectly regulating dopaminergic transmission.

In rodent models, KD has been shown to increase extracellular levels of GABA and agmatine—both inhibitory neuromodulators—without significantly altering glutamate concentrations. Calderón et al. (2017) found that hippocampal levels of GABA and agmatine were significantly elevated in rats maintained on KD, while glutamate remained stable, suggesting a shift toward inhibitory dominance​. Similarly, Włodarczyk et al. (2018) proposed that KD may support schizophrenia treatment by enhancing the GABA:glutamate ratio, potentially counteracting the GABAergic deficits observed in the disorder.

KD also suppresses presynaptic glutamate release via vesicular mechanisms. Juge et al. (2010) showed that ketone bodies, such as acetoacetate, compete with chloride ions at vesicular glutamate transporters (VGLUTs), thereby decreasing vesicular glutamate loading and release, which could reduce excitotoxicity and aberrant cortical signaling​.

Although direct evidence for dopaminergic modulation by KD in schizophrenia is limited, several indirect mechanisms suggest potential relevance. Notably, KD increases brain levels of adenosine (Ruskin et al., 2025)​, a neuromodulator that inhibits dopamine release via activation of A1 receptors (Rogawski et al., 2016)​. This adenosine-mediated dampening of dopaminergic tone could be especially beneficial in mitigating the hyperdopaminergic states associated with positive symptoms, as well as improving cognitive and affective regulation (Howes et al., 2024)​.

In the prefrontal cortex, KD selectively enhances inhibitory signaling. Hernandez et al. (2018) found that KD-fed rats exhibited increased expression of vesicular GABA transporters (VGAT), without corresponding changes in vesicular glutamate transporters (VGLUT1). These biochemical adaptations correlated with improved cognitive flexibility and reduced anxiety-like behavior​, aligning with the cognitive and emotional impairments frequently seen in schizophrenia.

Moreover, mitochondrial function may also contribute to KD’s neuromodulatory effects. Kwon et al. (2016) demonstrated that presynaptic mitochondria regulate calcium clearance, which in turn controls neurotransmitter release. By enhancing mitochondrial efficiency, KD may support tighter regulation of calcium-dependent synaptic transmission, promoting circuit stability and homeostasis​.

### 4.3 Modulation of neuroinflammation

An expanding body of evidence implicates chronic low-grade neuroinflammation as a key contributor to the pathophysiology of schizophrenia. Altered immune signaling—characterized by elevated levels of proinflammatory cytokines and activated microglia—has been documented in both postmortem brain tissue and *in vivo* studies. Prenatal maternal immune activation (MIA), systemic inflammation, and dysregulated microglial function are thought to interfere with neurodevelopmental processes, ultimately contributing to the emergence of cognitive, affective, and psychotic symptoms (Chaves et al., 2024).

Nutritional ketosis may modulate several neuroinflammatory pathways implicated in schizophrenia. Elevated concentrations of cytokines such as interleukin-6 (IL-6), tumor necrosis factor-alpha (TNF-α), and interleukin-1 beta (IL-1β) have been repeatedly observed in individuals with schizophrenia (Chaves et al., 2024). In a pilot study involving patients with refractory temporal lobe epilepsy, intermittent ketogenesis induced by medium-chain triglyceride supplementation significantly reduced levels of these same cytokines, while also increasing regulatory T cells (Khatami et al., 2025). These findings suggest that the anti-inflammatory effects of ketogenesis may extend beyond epilepsy and hold promise for other neuropsychiatric conditions marked by persistent inflammation.

Supporting this, Monda et al. (2024) reviewed extensive preclinical evidence showing that the ketogenic diet (KD) attenuates neuroinflammation by modulating astrocytic and microglial responses, lowering proinflammatory cytokines (including IL-1β, IL-6, and TNF-α), and fostering a more neuroprotective immune milieu. These effects are especially relevant to schizophrenia, where excessive microglial activation and aberrant cytokine release have been implicated in abnormal synaptic pruning and cortical thinning (Chaves et al., 2024).

Microglial polarization appears to be a key mechanism through which KD exerts its immunomodulatory effects. Ji and Tang (2025) demonstrated that nutritional ketosis promotes an anti-inflammatory microglial phenotype by inhibiting key inflammatory pathways, including NF-κB, MAPK, and the NLRP3 inflammasome. Such modulation may help mitigate synaptic toxicity and neuroinflammation, both of which are increasingly recognized as contributing factors in schizophrenia.

In addition to its immunological effects, KD may also confer neuroprotection through redox modulation. The ketone body β-hydroxybutyrate functions not only as an alternative energy substrate but also as a signaling molecule with epigenetic effects. BHB inhibits class I and IIa histone deacetylases (HDACs), leading to the upregulation of antioxidant genes such as SOD2, catalase, and FOXO3a (Miller et al., 2018). These changes support redox homeostasis and may protect neurons in metabolically demanding brain regions that are especially vulnerable to oxidative stress.

## 5 Potential clinical applications of ketogenic therapy in schizophrenia

The growing body of evidence supporting the metabolic and neuropharmacological effects of ketogenic therapy in schizophrenia suggests several potential clinical applications. While current data are preliminary, they indicate the possibility of targeted interventions for symptom domains poorly addressed by standard antipsychotic treatments, as well as a broader role in addressing the physical health burden associated with the illness. In parallel, new hypotheses emerge regarding early intervention and personalized treatment strategies. This section outlines key potential applications and research priorities to guide future investigation.

### 5.1 Targeting negative symptoms and cognitive dysfunction

Negative symptoms and cognitive deficits are among the most debilitating and treatment-resistant aspects of schizophrenia. These include avolition, anhedonia, social withdrawal, and impairments in executive function, working memory, and attention. Current pharmacological treatments offer limited efficacy in addressing these domains, underscoring the need for alternative therapeutic strategies (Correll and Schooler, 2020; Feber et al., 2025; Horan et al., 2025). Emerging data suggest that ketogenic therapy may offer unique advantages by modulating neural metabolism, synaptic transmission, and inflammatory signaling.

Numerous studies have shown that ketogenic therapy improve cognitive function across various clinical and preclinical populations. A systematic review by Chinna-Meyyappan et al. (2023) reported that over 80% of human studies assessing cognition after ketogenic interventions demonstrated positive effects, specifically in executive functioning, working memory, and attention–areas of cognition known to be particularly impacted in schizophrenia (Heinrichs and Zakzanis, 1998)​. These findings are supported by a meta-analysis of randomized controlled trials in Alzheimer’s disease, which showed significant improvements in MMSE, ADAS-Cog, and global mental state scores following ketogenic interventions (Rong et al., 2024)​.

In preclinical models, Wang et al. (2021) demonstrated that nutritional ketosis improved spatial reference memory in rats with pentylenetetrazol (PTZ)-induced cognitive impairment. Mechanistically, nutritional ketosis restored the expression of key synaptic proteins, such as GluR1 and NR2B, and normalized overactivation of the MAPK signaling pathway​. Similarly, Hernandez et al. (2018) showed that KD-fed aged rats exhibited improved performance in tasks requiring prefrontal–hippocampal interaction, such as spatial alternation and working memory/bi-conditional association tasks. These behavioral effects were associated with increased expression of ketone transporters (MCT1, MCT4) and GABAergic markers in the prefrontal cortex (Hernandez et al., 2018)​.

These findings are mechanistically relevant to schizophrenia, where altered energy metabolism, excitatory–inhibitory imbalance, and mitochondrial dysfunction are strongly implicated in cognitive and negative symptoms. KD’s ability to restore mitochondrial efficiency and provide ketone-fueled ATP production may compensate for prefrontal hypometabolism observed in schizophrenia, supporting higher-order cognitive processing through improved synaptic stability and neurotransmitter balance.

As previously mentioned, several preclinical studies showed that a ketogenic diet reversed social withdrawal and working memory deficits induced by NMDA receptor hypofunction (Table 2). Consistent with these findings, clinical case reports have also documented improvements in both negative and cognitive symptoms under ketogenic therapy, including cases of schizoaffective disorder with sustained remission and functional recovery (Palmer, 2017; Laurent et al., 2025).

Beyond synaptic mechanisms, recent work by Minhas et al. (2021) showed that rejuvenating energy metabolism in aged myeloid cells reverses cognitive decline, highlighting the link between systemic inflammation, energy metabolism, and cognition​. This is particularly relevant in schizophrenia, where chronic inflammation and impaired glucose utilization coexist.

In summary, while direct evidence in schizophrenia remains preliminary, converging data from animal models, mechanistic studies, and clinical reports suggest that ketogenic therapy may hold promise for targeting negative symptoms and cognitive dysfunction. These effects appear to be mediated through enhanced synaptic plasticity, mitochondrial efficiency, anti-inflammatory signaling, and improved neurotransmitter balance.

### 5.2 Early intervention in high-risk populations

The onset of schizophrenia is often preceded by a prodromal phase characterized by subclinical symptoms, including cognitive deficits, social withdrawal, and attenuated psychotic features. Early intervention strategies have traditionally focused on psychosocial and pharmacological approaches. However, access to psychological intervention is often limited and many patients and families are reluctant to engage in medication treatment. Emerging evidence suggests that metabolic dysfunction—notably insulin resistance and impaired glucose homeostasis—may be present even before the onset of full-blown psychosis, highlighting the potential for ketogenic therapy as an early disease-modifying intervention that may be more appealing to young patients and families.

#### 5.2.1 Evidence of early metabolic disturbance

As mentioned above, several studies have reported significant alterations in glucose metabolism and insulin resistance among individuals with first-episode, antipsychotic-naïve schizophrenia. A meta-analysis by Pillinger et al. (2017) demonstrated elevated fasting glucose, insulin levels, and HOMA-IR scores in this population compared to controls​. Steiner et al. (2017) further confirmed these findings, identifying a twofold increase in insulin resistance and elevated cortisol levels among drug-naïve patients​. These metabolic disturbances appear to precede antipsychotic exposure and are not entirely attributable to lifestyle or stress-related confounders.

Furthermore, Hackinger et al. (2018) provided compelling evidence for a shared genetic predisposition to schizophrenia and type 2 diabetes, identifying 29 genes with overlapping associations and several colocalizing genomic loci​. This shared genetic vulnerability reinforces the notion that metabolic dysfunction is not merely a consequence of illness progression or medication but may play a pathogenic role from the earliest stages.

#### 5.2.2 Schizotypy traits and ketogenic therapy

The continuum model of psychosis suggests that schizophrenia-spectrum traits are distributed in the general population, with schizotypy reflecting early vulnerability. In a non-clinical sample, Garner et al. (2024) found that individuals following a ketogenic diet exhibited significantly lower positive and negative schizotypy traits—including magical thinking, unusual perceptual experiences, constricted affect, and social anxiety—compared to those on other diets​. These findings suggest that the ketogenic diet may modulate risk factors even at subclinical levels, potentially offering prophylactic benefits.

#### 5.2.3 Safety and feasibility in pediatric and young adult populations

The ketogenic diet has a long-standing safety record in pediatric populations, distinctly for treatment-resistant epilepsy. Omori et al. (2024) highlighted the growing interest in KD for various neurodevelopmental disorders such as ADHD and ASD, suggesting it may be well tolerated in children and adolescents when appropriately monitored​. Though clinical studies in youth at high risk for psychosis are still lacking, these safety data support the rationale for exploring ketogenic interventions during critical neurodevelopmental windows.

#### 5.2.4 Rationale for early intervention

Given the early onset of metabolic alterations and the potential neuroprotective effects of ketone bodies—including anti-inflammatory action, improved mitochondrial efficiency, and stabilization of neuronal excitability—the ketogenic diet may offer a unique opportunity to intervene before irreversible neurodegenerative changes occur. Chrysafi et al. (2024) emphasized the need for future randomized controlled trials to evaluate KD as both a preventive and adjunctive treatment in psychiatric conditions, including schizophrenia​.

### 5.3 Adjunctive treatment for super-refractory schizophrenia

Super-refractory or clozapine-resistant schizophrenia (CRS) refers to the persistence of disabling symptoms—positive, negative, or cognitive—despite an adequate trial of clozapine, which remains the gold standard for treatment-resistant schizophrenia (i.e., persistence of symptoms despite adequate trials of at least two different antipsychotics). It is estimated that up to 60% of patients with treatment-resistant schizophrenia do not achieve meaningful remission with clozapine monotherapy, placing them in the super-refractory category (Campana et al., 2021; Chakrabarti, 2021)​​.

Despite the widespread use of pharmacological augmentation strategies—explicitly antipsychotic polypharmacy—systematic reviews and meta-analyses show only small, inconsistent, and often clinically insignificant benefits. Wagner et al. (2021) highlighted that, even among expert consensus, the strongest support was reserved for interventions like aripiprazole or amisulpride augmentation, electroconvulsive therapy (ECT), and psychosocial support, yet even these lacked robust evidence from high-quality randomized controlled trials​. Non-pharmacological strategies like Cognitive Behaviour Therapy for Psychosis (CBTp) and repetitive Transcranial Magnetic Stimulation (rTMS) have shown limited and mixed results​​ (Campana et al., 2021; Chakrabarti, 2021).

Given this critical unmet need, novel therapeutic approaches targeting underlying pathophysiological mechanisms are urgently required. The ketogenic therapy may be a potential adjunctive strategy for patients with super-refractory schizophrenia, based on several lines of evidence.

1. Mechanistic Rationale Beyond Dopamine: Traditional pharmacotherapies largely target dopamine receptors. KD, in contrast, influences metabolic pathways, mitochondrial function, glutamatergic transmission, and neuroinflammation—all of which are dysregulated in schizophrenia and may not be directly addressed by clozapine or its augmenting agents (Sarnyai et al., 2019).
2. Case Reports and Clinical Observations: Recent clinical reports have shown that patients with long-standing, treatment-resistant schizophrenia—including those on clozapine—have achieved marked improvements in both positive and negative symptoms after initiating a ketogenic diet (Palmer, 2017; Sarnyai et al., 2019)​.
3. Dual Benefit: Psychiatric and Metabolic Outcomes: Patients on clozapine and other atypical antipsychotics often develop significant metabolic comorbidities, including insulin resistance, weight gain, and dyslipidemia. The ketogenic diet has shown promise in addressing these adverse effects, potentially improving overall health outcomes and enhancing symptomatic management. Its potential to improve both domains makes it a singularly attractive candidate for patients with CRS.
4. Personalized and Multimodal Approach: As emphasized by Chakrabarti (2021), future strategies for CRS must go beyond simple pharmacological add-ons. The KD fits within a personalized and multimodal treatment paradigm, potentially offering individualized metabolic support to patients for whom conventional strategies have failed.

While evidence remains limited to case reports and small series, the low-risk profile of KD, its well-documented safety in epilepsy populations, and its mechanistic plausibility justify pilot trials in CRS populations. Careful attention to feasibility, nutritional supervision, and integration with psychosocial supports will be essential for successful implementation.

### 5.4 Metabolic syndrome management

Individuals with schizophrenia face an alarmingly elevated risk of premature mortality, with all-cause mortality rates nearly 2.5 to 3.7 times higher than in the general population (Saha et al., 2007; Olfson et al., 2015). A recent meta-analysis involving over 2.7 million individuals confirmed this excess mortality in both males and females, with minimal sex differences (Solmi et al., 2025). Cardiovascular disease stands out as the leading cause of death in this population, driven by a constellation of modifiable risk factors such as obesity, dyslipidemia, insulin resistance, and type 2 diabetes mellitus (Saha et al., 2007; Olfson et al., 2015). These factors often converge under the umbrella of metabolic syndrome, a condition disproportionately prevalent among patients with schizophrenia, especially those receiving second-generation antipsychotics like olanzapine.

Antipsychotic-induced metabolic dysfunction has become a critical concern in the treatment of schizophrenia. The metabolic side effects of antipsychotics are not limited to weight gain and dyslipidemia but also include rapid and significant alterations in glucose homeostasis. Preclinical studies demonstrate that a single dose of olanzapine can acutely elevate blood glucose levels in mice (Shamshoum et al., 2022), an effect mediated by increased serum glucagon and independent of insulin resistance. Importantly, fasting or short-term ketogenic diet—both of which raise circulating ketone body levels—were sufficient to prevent olanzapine-induced hyperglycemia in these animal models (Budd and MacIntyre, 2022; Shamshoum et al., 2022). These findings highlight the potential for ketogenic interventions to buffer the acute glycemic dysregulation caused by second-generation antipsychotics.

In addition to protective effects against glucose dysregulation, ketogenic diets may also mitigate long-term metabolic complications. A growing body of evidence supports the beneficial impact of ketogenic interventions on body weight, lipid profiles, and insulin sensitivity, even in individuals with serious mental illness. For instance, ketogenic nutritional strategies have been associated with reductions in triglyceride levels, improvements in HDL cholesterol, and lower fasting glucose and insulin levels (Biesiekierska et al., 2025). These metabolic improvements are peculiarly meaningful given the challenges in managing lifestyle-related risk factors in schizophrenia due to cognitive impairment, negative symptoms, and limited access to healthcare.

Given that metabolic dysfunction is a key contributor to the excess mortality in schizophrenia (Olfson et al., 2015), therapeutic strategies that target both psychiatric and metabolic dimensions are urgently needed. The ketogenic diet offers a rare dual benefit—it may improve core psychiatric symptoms while simultaneously addressing the somatic comorbidities that drive premature mortality. Future clinical studies should assess the feasibility, safety, and sustainability of ketogenic dietary interventions as adjunctive treatments in schizophrenia, particularly in individuals at high risk for metabolic syndrome or those already exhibiting metabolic complications.

## 6 Discussion

This article reviewed the emerging evidence and rationale supporting ketogenic therapy as a novel intervention in schizophrenia. While traditionally conceptualized as a disorder of neurotransmission, schizophrenia is now increasingly understood to involve profound alterations in systemic and cerebral metabolism—especially impairments in glucose utilization, mitochondrial function, and brain energy availability (Pillinger et al., 2017; Holper et al., 2019; Sullivan et al., 2019). These metabolic vulnerabilities are detectable even at early illness stages and are compounded by the adverse cardiometabolic effects of second-generation antipsychotics (Olfson et al., 2015; Steiner et al., 2017).

Ketogenic therapy, by shifting cerebral metabolism from glucose to ketone bodies, provides an alternative and efficient fuel source for the brain. Preclinical and translational studies demonstrate that ketone bodies such as β-hydroxybutyrate can restore mitochondrial function, increase ATP production, modulate neurotransmission, and reduce neuroinflammation and oxidative stress (Miller et al., 2018; Kyriazis et al., 2022; Ji and Tang, 2025). These neurobiological effects align closely with known pathophysiological features of schizophrenia—including hypofrontality, excitatory/inhibitory imbalance, and elevated inflammatory markers—supporting the plausibility of ketogenic interventions in this context (Du et al., 2014; Rowland et al., 2016; Hernandez et al., 2018).

Clinical observations and early case reports suggest that ketogenic therapy may offer symptom improvement in individuals with schizophrenia spectrum disorders, expressly in domains that remain refractory to standard pharmacological treatment. These include negative symptoms, cognitive dysfunction, and persistent symptoms in super-refractory patients (Palmer, 2017; Sarnyai et al., 2019). Additionally, the diet’s capacity to improve insulin sensitivity, reduce weight, and attenuate antipsychotic-induced hyperglycemia offers a rare dual benefit: targeting both psychiatric symptoms and physical health comorbidities that contribute to early mortality (Saha et al., 2007; Shamshoum et al., 2022; Biesiekierska et al., 2025).

However, caloric restriction may confound some of these observed benefits. While the ketogenic diet is effective for weight loss (Baylie et al., 2024), several studies suggest that its therapeutic effects extend beyond reductions in body weight. For instance, Dietch et al. (2023) observed significant mood improvements within 4 days of KD initiation—well before meaningful weight loss. In animal models of schizophrenia, Kraeuter et al. (2019b) and Tregellas et al. (2015) demonstrated behavioral improvements independent of weight change. Similarly, in two clinical cases of schizoaffective disorder, Laurent et al. (2025) reported symptom remission with minimal or no weight loss. These findings point to ketosis-specific mechanisms as the main driver of clinical effects, independently of caloric restriction.

Emerging evidence also suggests that ketogenic interventions may hold potential in younger or high-risk populations. Given the early emergence of metabolic alterations—sometimes preceding full-blown psychosis—ketogenic therapy may offer a neuroprotective, disease-modifying strategy in the prodromal phase that may be more acceptable to young patients and their families (Hackinger et al., 2018; Garner et al., 2024). Its well-established safety in pediatric epilepsy and growing interest in its use across neurodevelopmental conditions support further exploration in these groups (Omori et al., 2024).

Collectively, these findings support the potential of KD as a novel intervention for several unmet therapeutic needs in schizophrenia, including persistent negative symptoms and cognitive dysfunction, prevention strategies in high-risk populations, metabolic syndrome management, and adjunctive therapy in super-refractory cases. Notably, KD may also mitigate some of the adverse metabolic effects associated with antipsychotic use, potentially improving both psychiatric and physical health outcomes (Norwitz et al., 2020).

## 7 Limitations

Despite the promising mechanistic rationale and emerging clinical observations, several limitations must be acknowledged. First, the current evidence base remains limited to preclinical studies, case reports, and small open-label trials. To date, there are no large-scale randomized controlled trials evaluating ketogenic therapy in schizophrenia, limiting the generalizability and strength of clinical recommendations.

Second, adherence to a strict ketogenic diet may be challenging for individuals with schizophrenia, particularly in the context of negative symptoms, cognitive impairment, and socioeconomic barriers. Most published cases involved highly motivated participants with strong support systems, which may not reflect broader psychiatric populations.

Third, long-term safety and sustainability of ketogenic therapy in schizophrenia remain unclear. While the diet has an established safety profile in pediatric epilepsy and metabolic disorders, its effects over extended periods in adults with serious mental illness—especially those taking antipsychotics—have not been adequately studied.

Fourth, heterogeneity in ketogenic interventions (e.g., classical KD, modified Atkins, exogenous ketones), outcome measures, and patient characteristics across existing studies limits comparability. Standardized protocols and biomarker-driven stratification will be essential for future trials to determine who is most likely to benefit and under what conditions.

Finally, while several mechanistic pathways have been proposed to explain how ketogenic therapy may exert therapeutic effects—such as modulation of GABA/glutamate balance, anti-inflammatory properties, and mitochondrial enhancement—much of this evidence is extrapolated from epilepsy models or general neuroscience studies. Direct evidence for these mechanisms in schizophrenia populations remains scarce. For example, studies demonstrating changes in brain metabolism, mitochondrial function, or inflammatory markers following ketogenic interventions in patients with schizophrenia are currently lacking. As such, while these pathways offer promising theoretical grounding, they should be interpreted cautiously until validated by disorder-specific empirical research.

## 8 Future research

Future studies should address methodological variability in current clinical trials of ketogenic therapy. Many assume ketosis results from dietary intake alone, yet caloric consumption is often poorly reported, raising the possibility that starvation physiology may confound outcomes. Hunger can also affect adherence, and the lack of macronutrient characterization complicates interpretation of metabolic effects. Effective counselling—before and during treatment—tailored to the patient’s clinical, psychological, and social context, is key to improving adherence (Kalra et al., 2019).

To move forward, future research should also prioritize well-designed randomized controlled trials to assess the efficacy of ketogenic therapy across symptom domains, especially negative and cognitive symptoms. Consistent use of validated outcome measures—such as the PANSS, MCCB, and standardized metabolic assessments—will improve comparability and facilitate meta-analyses. Studies should also investigate disorder-specific mechanisms by incorporating accessible biomarkers, such as peripheral cytokines, oxidative stress markers, and mitochondrial function assays, which are increasingly recognized as proxies for central nervous system alterations in schizophrenia. While direct *in vivo* measurement of neurotransmitter levels remains challenging, emerging neuroimaging techniques such as magnetic resonance spectroscopy offer promising insights into neurometabolic effects. Incorporating such biomarkers may help clarify mechanisms and identify individuals most likely to benefit.

The growing number of registered and ongoing clinical trials investigating ketogenic therapy for schizophrenia and related disorders (Table 3) reflects increasing interest in the field. However, these studies vary in design, populations, and endpoints, and many remain in early stages or lack mechanistic depth. This underscores the continued need for well-conceived studies that not only evaluate clinical outcomes but also incorporate biomarker assessment, track adherence, and explore translational strategies applicable to diverse psychiatric care settings.

**TABLE 3:** Upcoming and ongoing trials of ketogenic therapy in schizophrenia and related disorders.

Trial ID	Title	Design	Masking	Primary Outcomes	Duration (weeks)	Protocol Notes	Principal Investigator(s)	Estimated Primary Completion
NCT05268809	Can Neural Network Instability in Schizophrenia Be Improved with a Ketogenic Diet?	RCT (parallel groups)	None (open-label)	Neural network instability, metabolic and inflammatory markers	4	Ketogenic vs. Diet as usual; meals delivered; advanced 7T fMRI	Ford, J	Aug 2025
NCT05968638	Ketogenic Diet in People With Schizophrenia	RCT (parallel groups)	Investigator and outcomes assessor blinded; participants unblinded	Psychiatric and metabolic outcomes (BPRS, metabolic markers)	12	Ketogenic vs. regular diet; inpatient	Kelly, D	Aug 2026
NCT03873922	Ketogenic Diet for Psychotic Disorders	RCT (parallel groups)	Investigator and outcomes assessor blinded; participants unblinded	PANSS, feasibility and tolerability	6	Modified KD in psychotic inpatients	Ruusunen, A	Jun 2025
NCT06221852	Ketogenic and Nutritional Interventions for First Episode Bipolar Disorder and Psychosis	RCT (parallel, metabolic/neuroimaging study)	None (open-label)	Brain NAD+/NADH, PANSS, HAM-D, insulin resistance	12	Early intervention study using neuroimaging and blood markers	Chouinard, V.-A	Sep 2027
NCT06558201	Open Label Extension Study of NCT06221852	Single-arm extension study	Open-label extension	Metabolic, psychiatric, and neuroimaging measures	12	12-week continuation of initial trial	Chouinard, V.-A	Sep 2027
ACTRN12623000854639	Ketogenic Metabolic Therapy in Schizophrenia and Bipolar Disorder	RCT (parallel groups with active comparator)	Outcome assessor and analyst-blinded; participants know diet type	PANSS, cognition (CANTAB), metabolic markers	14	Ketogenic diet vs. healthy diet with dietitian support. Protocol published by Longhitano et al. (2024)	Sarnyai, Z	Dec 2024
NCT06748950	Ketogenic Diet in Serious Mental Illness: Deep Omic Profiling	RCT (parallel groups with crossover)	Open-label with crossover	WHO-5, BPRS, metabolic/omic profiling	24	12-week ketogenic or diet as usual with crossover	Sethi, S	Mar 2028

### 8.1 Implementation considerations and adherence challenges

While ketogenic therapy offers a promising, mechanistically informed approach to schizophrenia treatment, its feasibility in real-world psychiatric settings may be constrained by practical challenges. Individuals with schizophrenia frequently experience cognitive impairment, negative symptoms, and functional limitations that can interfere with the demands of dietary interventions like ketogenic therapy. Common challenges include difficulties with meal preparation, financial constraints, subclinical symptoms, and limited caregiver support.

Table 4 summarizes key barriers to implementation and offers context-adapted strategies to improve feasibility in neuropsychiatric settings. These strategies include budget-conscious meal planning, simplified protocols, family involvement, and digital support tools. Models adapted from pediatric epilepsy care—such as training cohabiting relatives—may be particularly valuable (Fabe et al., 2018). Self-management plans—already embedded in case management models—can include simplified ketogenic meal planning, with occupational therapy supporting adherence, particularly for patients with cognitive challenges.

**TABLE 4:** Practical challenges and proposed Solutions for implementing ketogenic therapy in schizophrenia.

Challenge	Description	Proposed Solution
Lack of motivation or initiative	Negative symptoms may limit motivation and goal-directed behavior, reducing engagement with complex dietary plans	Use SMS reminders, motivational interviewing, and brief support strategies to foster routine and autonomy
Meal preparation difficulties	Cognitive impairment and disorganization may interfere with meal planning and cooking tasks	Offer meal delivery options or ready-to-eat ketogenic kits through health programs or caregivers
Need for caregiver involvement	Patients often depend on family or support persons to manage dietary adherence and daily routines	Train cohabiting family members to assist with meal prep and monitor adherence, adapting pediatric models
Dropout due to perceived inefficacy or effort	Patients may stop therapy if improvements are not immediate or if the effort is perceived as too demanding	Set achievable expectations, personalize goals, and use peer support or early feedback to sustain motivation
Subclinical symptoms	Residual symptoms, including cognitive deficits and low insight, often undermine treatment adherence	Caregiver support and interprofessional team with OT may help meal planning as part of self-management plan
Perceived social restrictions	Diet restrictions can create social isolation or interfere with participation in meals and celebrations	Include culturally appropriate meals and educate peers to foster understanding and reduce stigma
Side effects and dietary tolerability	Constipation, weight fluctuations, or metabolic issues may lead to reduced tolerability and early discontinuation	Adopt gradual initiation protocols, monitor early side effects, and provide responsive clinical guidance
Cost of groceries	Financial limitations can restrict access to nutrient-dense ketogenic ingredients and specialty items	Adapt menus to low-cost ketogenic staples and provide budgeting tips for diet sustainability
Limited access to trained professionals	A shortage of trained professionals may limit access to supervised ketogenic interventions in psychiatry	Expand telehealth access, train interdisciplinary teams, and develop toolkits for psychiatric settings
Time required for preparation and monitoring	Frequent calculations and time-consuming food prep can overwhelm patients and caregivers	Leverage digital apps, exchange lists, and modular tools to ease planning and support time efficiency
Need for long-term supervision	Many patients require continuous support and cannot manage the ketogenic diet independently	Implement ongoing education and accessible contact with the clinical team to build long-term adherence

Long-term adherence poses additional challenges. Insights from the Glut1 Deficiency Collective Voices Project, which surveyed over 260 individuals across 31 countries, highlight common barriers to sustained KT use: side effects, social restrictions, time demands, lack of trained professionals, and food-related misconceptions (Foundation, 2022). Surprisingly, while 82% of respondents stated that benefits outweighed the difficulties, 80% of adult patients reported an inability to maintain the diet without structured supervision. These findings reinforce the need for ongoing clinical support, patient and caregiver education, and sustained guidance.

Implementation strategies developed in the epilepsy field also offer useful models for psychiatric populations. In particular, the “low and slow outpatient” approach—characterized by gradual dietary initiation and close outpatient monitoring—suggest minimal to no side effects and very high patient sustainability to the therapy (Fabe and Ronen, 2018). This slower approach allows for patients and families to have a positive initiation experience and more time to incorporate the new KD lifestyle required while being supervised by the medical KD team (Fabe et al., 2018).

Initiation and maintenance methodologies, distinguishing the tasks between the patient and the supervising clinician is also undoubtedly essential for enhancing compliance and long term adherance. Food exchange lists, meal plans, KD recipes and different nutritional modulars (e.g., MCT oil, fat powders) have been well established as tools used in the KD practice. Additional KD maintenance tools are fast in development in the technology sector such KD digital platforms that can be used by the clinician and patient under supervised conditions to ease some of the practical calculations and offer creative menu options–e.g., Ketosuite (Canada, New Zealand) and Ketogenic Mealplanner (UK) (Tan-Smith, 2023).

Future clinical trials of ketogenic diet in schizophrenia should incorporate these practical elements, assess their impact on adherence and outcomes, and evaluate their feasibility in real-world psychiatric settings. Embedding feasibility measures—such as recruitment rates, retention, acceptability, and implementation fidelity—can provide critical insights into the practicality and scalability of ketogenic interventions. Real-world implementation studies that prioritize both clinical and feasibility outcomes will be essential to ensure that promising neurobiological mechanisms can translate into sustainable, patient-centered interventions.

## 9 Conclusion

Schizophrenia is increasingly recognized as a disorder of disrupted bioenergetics, immune activation, and synaptic imbalance—factors that are not adequately addressed by current pharmacological treatments. Ketogenic therapy offers a metabolically grounded, mechanistically plausible, and potentially transformative approach that targets these core dysfunctions. By improving mitochondrial function, modulating neurotransmission, and reducing inflammation, ketogenic interventions may offer benefits across multiple symptom domains, including cognition, negative symptoms, and treatment resistance, while also mitigating antipsychotic-induced metabolic burden. Although clinical evidence remains preliminary, the convergence of pathophysiological insights and early therapeutic signals provides a compelling foundation for rigorous investigation. If proven effective, ketogenic therapy could represent a significant step toward a more integrative and personalized treatment paradigm in schizophrenia.
